# Induction of p53 Phosphorylation at Serine 20 by Resveratrol Is Required to Activate p53 Target Genes, Restoring Apoptosis in MCF-7 Cells Resistant to Cisplatin

**DOI:** 10.3390/nu10091148

**Published:** 2018-08-23

**Authors:** Jorge Hernandez-Valencia, Enrique Garcia-Villa, Aquetzalli Arenas-Hernandez, Jaime Garcia-Mena, Jose Diaz-Chavez, Patricio Gariglio

**Affiliations:** 1 Departamento de Genética y Biología Molecular, Centro de Investigación y de Estudios Avanzados (CINVESTAV-IPN), Av. IPN No. 2508, Gustavo A. Madero, Ciudad de México 07360, Mexico; jhernandezv@cinvestav.mx (J.H.-V.); liebre1963@yahoo.com (E.G.-V.); aquetzalliarenas@gmail.com (A.A.-H.); jgmena@cinvestav.mx (J.G.-M.); 2 Unidad de Investigación Biomédica en Cáncer, Instituto de Investigaciones Biomédicas, UNAM/Instituto Nacional de Cancerología, Av. San Fernando No. 22, Sección XVI, Tlalpan, Ciudad de México 14080, Mexico

**Keywords:** breast cancer, cisplatin, p53, phosphorylation, resistance, resveratrol

## Abstract

Resistance to cisplatin (CDDP) is a major cause of cancer treatment failure, including human breast cancer. The tumor suppressor protein p53 is a key factor in the induction of cell cycle arrest, DNA repair, and apoptosis in response to cellular stimuli. This protein is phosphorylated in serine 15 and serine 20 during DNA damage repair or in serine 46 to induce apoptosis. Resveratrol (Resv) is a natural compound representing a promising chemosensitizer for cancer treatment that has been shown to sensitize tumor cells through upregulation and phosphorylation of p53 and inhibition of RAD51. We developed a CDDP-resistant MCF-7 cell line variant (MCF-7_R_) to investigate the effect of Resv in vitro in combination with CDDP over the role of p53 in overcoming CDDP resistance in MCF-7_R_ cells. We have shown that Resv induces sensitivity to CDDP in MCF-7 and MCF-7_R_ cells and that the downregulation of p53 protein expression and inhibition of p53 protein activity enhances resistance to CDDP in both cell lines. On the other hand, we found that Resv induces serine 20 (S20) phosphorylation in chemoresistant cells to activate p53 target genes such as *PUMA* and *BAX*, restoring apoptosis. It also changed the ratio between BCL-2 and BAX, where BCL-2 protein expression was decreased and at the same time BAX protein was increased. Interestingly, Resv attenuates CDDP-induced p53 phosphorylation in serine 15 (S15) and serine 46 (S46) probably through dephosphorylation and deactivation of ATM. It also activates different kinases, such as CK1, CHK2, and AMPK to induce phosphorylation of p53 in S20, suggesting a novel mechanism of p53 activation and chemosensitization to CDDP.

## 1. Introduction

Cisplatin (CDDP) is an anticancer drug for the treatment of various types of cancer including human breast cancer. CDDP mediates its anticancer effect by inhibition of DNA synthesis or by saturation of the cellular capacity to repair platinum adducts of DNA. However, resistance to CDDP is a major cause of treatment failure, and the molecular mechanisms are poorly understood [1].

Due to this phenomenon, it is necessary to continue the search for effective chemosensitizers for cancer treatment. One promising possibility is the use of natural compounds like resveratrol (Resv), which is a phytoalexin present in extracts of more than 70 plant species with a broad spectrum of beneficial health effects including anticancer functions. The reported anticancer activities of Resv are mediated through the modulation of several cell signaling molecules that regulate cell cycle progression, proliferation, apoptosis, invasion, metastasis, and angiogenesis of tumor cells. Although not fully understood, most of the activities of Resv are due to the presence of a phenol and m-hydroquinone moieties, especially the 4-hydroxyl group of the phenol ring which has been attributed with scavenging of free radicals, inhibition of proliferation, and genotoxic activity [2,3,4,5]. Resv can sensitize resistant cells to chemotherapeutic agents, including CDDP, by overcoming one or more mechanisms of chemoresistance [6,7]. Evidence suggests that the downregulation of the wild-type p53 tumor suppressor protein enhanced tumor cell survival, conferring a mechanism of chemoresistance [8]. However, in a few cases, Resv has been shown to sensitize tumor cells to chemotherapeutic agents through p53 dependent [9,10] or p53 independent pathways [11,12].

p53 is a key transcriptional factor in the induction of cycle arrest, DNA repair, and apoptosis in response to cellular stimuli. Promoter preference of target genes is determined by modification status of the p53 protein since it has two critical roles in the decision of cell fate, stopping the cell cycle to repair damaged DNA or the causing induction of apoptotic cell death [13]. Once cells are exposed to genotoxic agents, p53 is phosphorylated at the N-terminal transactivation domain by several kinases, resulting in an increment of expression. Serine 15 (S15) is phosphorylated by ATM at an earlier inductive phase (<24 h), followed by ATR at a later steady-state phase (>24 h) [14,15], and serine 20 (S20) is phosphorylated by CHK1/2. Both phosphorylations enable p53 to escape from MDM2-mediated ubiquitination and degradation. Stabilized p53 transactivates its target genes promoting cell cycle arrest (e.g., *P21*) followed by DNA repair. Under severe DNA damage (>24 h), serine 46 (S46) is also sequentially phosphorylated to maintain the level of S46 phosphorylation by ATM [15], and other kinases such as HIPK2 in response to UV irradiation [16], and DYRK2 in response to adriamycin and UV irradiation [13]. The phosphorylation of S46 is necessary to induce p53-mediated apoptosis-related genes such as *PUMA*, *NOXA*, *BAX,* and *PIG3* [17,18,19] and transcriptional repression of genes such as *BCL-2* [8].

It has been described that MCF-7 breast cancer cells have a surface integrin (αVβ3) that works as a receptor for Resv. This receptor is linked to induction of ERK1/2 and phosphorylation of p53 in S15 and S20 by Resv leading to apoptosis [20,21]. Moreover, we previously reported that treatment of MCF-7 cells with Resv induces the downregulation of several genes related to mismatch repair, DNA replication, and homologous recombination, decreasing protein levels of the MRN complex (MRE11-NBS1-RAD50) which is part of the homologous recombination DNA repair pathway [22]. Indeed, we found that downregulation of RAD51 sensitizes MCF-7 cells to CDDP treatment [23]. However, it is of maximal importance to understand the molecular mechanisms by which Resv overcome chemoresistance in cancer cells, alone or in combination with chemotherapeutic agents (e.g., CDDP), to enhance treatment efficacy and reduce toxicity.

Considering the previously reported anticancer function of Resv and its chemosensitizer capacity as well as phosphorylation of p53 induced by Resv, in this work we developed a CDDP-resistant MCF-7 cell line variant (MCF-7_R_) and investigated the effect of Resv in vitro in combination with CDDP in MCF-7 and MCF-7_R_ cells, the role of p53 in CDDP resistance, the involvement of Resv in p53 phosphorylation, and the role of the p53 pathway for overcoming resistance in MCF-7_R_ cells.

## 2. Materials and Methods

### 2.1. Reagents and Antibodies

Cisplatin (CDDP), resveratrol (Resv), 3-(4,5-dimethylthiazol-2-yl)-2,5-diphenyltetrazolium bromide (MTT), pifithrin-α, VP-16 and monoclonal anti-β-actin-HRP were purchased from Sigma-Aldrich (St. Louis, MO, USA). The AMPK inhibitor Compound C (or dorsomorphin), the CK1 inhibitor D4476, the Chk2 inhibitor, anti-rabbit and anti-mouse secondary antibodies, mouse monoclonal anti-phospho-ATM (S1981), rabbit polyclonal anti-ATM, monoclonal anti-p53-HRP (DO-1), and monoclonal anti-BCL-2 were purchased from Santa Cruz Biotechnology (San Diego, CA, USA). Rabbit monoclonal anti-BAX-HRP was purchased from Abcam (Cambridge, UK). Rabbit polyclonal anti-phospho-p53 (S15, S20 and S46) were from Cell Signaling Technology (Beverly, CA, USA).

### 2.2. Cell Lines and Cell Culture

The MCF-7 human breast cancer cells (ATCC) and MCF-7_R_ cells were cultured in Dulbecco’s modified Eagle’s medium (DMEM) supplemented with 10% (*v*/*v*) fetal bovine serum, penicillin (100 U/mL), streptomycin (100 μg/mL), and amphotericin B (0.25 μ/mL) in a 5% CO_2_ incubator at 37 °C. Additionally, MCF-7_R_ cells were continuously cultured with 5.5 μM of CDDP. Resv and CDDP stock solutions were prepared at a concentration of 80 mM in absolute ethanol and DMSO, respectively. Both compounds were diluted in culture medium at the final concentration indicated in each experiment.

### 2.3. Generation of the CDDP-Resistant MCF-7 Cell Line Variant (MCF-7_R_)

The MCF-7 human breast cancer cells were cultured with an initial treatment of 2 μM of CDDP and maintained at this concentration for 45 days until the monolayer density of the surviving cells was ~85%. Cells were harvested and plated 24 h before the second treatment with 4 μM of CDDP. After 33 days under treatment the surviving cells’ monolayer density reached ~85%. Finally, cells were harvested and plated 24 h before the third treatment with 6 μM of CDDP. After 13 days under treatment the surviving cells monolayer density was ~85%. At concentrations >6.5 μM of CDDP the cells died or formed clusters that prevented the formation of a cell monolayer. To create a MCF-7_R_ cell bank, cells were seeded at a density of 2 × 10^5^ cells/dish in p100 cell culture dishes and were continuously cultured with 5.5 μM of CDDP until the cell monolayer density was ~85%. Cells were frozen at a density of 2 × 10^6^ cells/cryovial and stored in liquid nitrogen. At the same time, the parental cell line was grown, so that the passages necessary to create the resistant cell line variant were equal for both cell lines.

### 2.4. Silencing of p53 Expression in MCF-7 and MCF-7_R_ Cells by shRNA

The SureSilencing shRNA Plasmid Kit (SABiosciences Qiagen, Frederick, MD, USA) was used to create stable MCF-7 and MCF-7_R_ cell lines with a down-regulated expression of p53 (MCF-7 p53-shRNA and MCF-7_R_ p53-shRNA). Control cells received a non-effective scrambled sequence (MCF-7 Ctrl-shRNA and MCF-7_R_ Ctrl-shRNA). Lipofectamine 2000 (Invitrogen, Gaithersburg, MD, USA) was used for transfections according to the manufacturer’s protocol. Additionally, for obtaining stable clones, cells were selected post transfection using Geneticin (G418, Thermo Fisher Scientific, Somerset, NJ, USA). Cell clones were expanded, and p53 contents were tested by Western blot.

### 2.5. Cell Viability Assay

Cells were plated at a density of 2 × 10^5^ cells/dish in p60 cell culture dishes 24 h before the assay. Cells were treated with different concentrations of CDDP (5, 10, 20, 30, 40 and 50 μM) with or without Resv (100 μM) for 48h. At the end of the treatment period, the cells were incubated with MTT (0.5 mg/mL) for 30 min. The medium was removed and the synthesized formazan dye crystals were solubilized with 500 μL of acid isopropanol, and absorbance was measured at a 570-nm wavelength (Tecan’s Sunrise absorbance microplate reader, Tecan Group Ltd., Männedorf, Switzerland). The growth percentage was calculated using the number of control cells with vehicle as 100% at 48 h.

### 2.6. Western Blot

Cells were seeded at a density of 2 × 10^5^ cells/dish in p60 cell culture dishes 24 h before the treatment. After the corresponding treatment, cells were lysed with RIPA lysis buffer (150 mM NaCl, 0.5% sodium deoxycholate, 0.1% SDS, 50 mM Tris, pH 7.4), 1 × Complete Mini Protease Inhibitor Cocktail (Roche Diagnostics, Branchburg, NJ, USA) and 1 × Phosphatase Inhibitor Cocktail C (Santa Cruz Biotechnology, Dallas, TX, USA). The cell suspension was sonicated and the supernatants were collected by centrifugation. Briefly, equal amount protein was resolved on a SDS-10% (*w*/*v*) polyacrylamide gel (for ATM and BCL-2 proteins values were 6% and 16% *w*/*v*, respectively). Proteins were transferred to a nitrocellulose membrane (GE Healthcare, Madison, WI, USA). Membranes were blocked (room temperature, 1 h) with Tween 20 (0.05%, *v*/*v*; TBS-Tween 20) containing bovine serum albumin (5%; *w*/*v*), then incubated overnight at 4 °C with the corresponding primary antibodies, followed by 1 h incubation with secondary antibodies conjugated to horse radish peroxidase (HRP). Protein was detected by Super Signal West Pico Chemiluminescent Substrate (Thermo Fisher Scientific, Somerset, NJ, USA). Signal intensity was determined densitometrically using Image Lab software, version 5.1 from Bio-Rad Laboratories (Hercules, CA, USA). All quantified Western blot data were corrected for loading using the anti-α-actin blots. Western blot figures are representative of at least three independent experiments.

### 2.7. Real-Time RT-PCR

Cells were plated at a density of 2 × 10^5^ cells/dish in p60 cell culture dishes 24 h before the treatment. After the corresponding treatment, total RNA was isolated using TRIzol reagent (Invitrogen Life Technologies) as described elsewhere. Integrity of RNA was determined by agarose gel analysis and quantified using a NanoDrop instrument (Thermo Scientific NanoDrop One/One, Waltham, MA, USA). Reverse transcription of total RNA was performed using the First Strand cDNA Synthesis Kit (Thermo Fisher Scientific, Somerset, NJ, USA). Real-time RT-PCR was performed using SYBR Green master mix (Thermo Fisher Scientific, Somerset, NJ, USA) in a 7300 Real Time PCR System instrument (Applied Biosystems, Foster City, CA, USA). The specificity of each PCR was examined by the melting temperature profiles of the final products. Reactions were conducted in triplicate, and relative amounts of gene were normalized to Beta-2 microglobulin (B2M). The relative gene expression data were analyzed by the comparative CT method (2^−ΔΔCT^ method). Primers: *P21*, *PUMA*, *NOXA*, *BAX*, *PIG3,* and *B2M* were purchased from Integrated DNA Technologies (IDT, Skokie, IL, USA) and forward and reverse sequences are presented in Table S1.

### 2.8. Apoptosis Analysis

Cells were plated at a density of 2 × 10^5^ cells/dish in p60 cell culture dishes 24 h before the treatment. After treatment, apoptosis analysis was performed using the Alexa Fluor 488 AnnexinV/Dead Cell Apoptosis Kit (Invitrogen V13245). Briefly, the cells were harvested, washed with cold PBS, and resuspended in 100 µL of Annexin binding buffer (ABB). Cells then were centrifuged and resuspended again in ABB supplemented with Alexa Fluor 488 Annexin V and 1 μg/mL of propidium iodide (PI). Cells then were incubated at room temperature for 15 min and finally, resuspended in 400 μL of ABB. Cells were analyzed by flow cytometry at 530 nm and 575 nm in a FACSCalibur instrument. Data analysis was performed on 20,000 events with the Summit Software Version 4.3. (Beckman Coulter Inc., Fullerton, CA, USA).

### 2.9. Statistical Analysis

Results are expressed as the mean ± SD of at least three independent experiments. The IC_50_ values for CDDP were calculated by nonlinear regression (curve fit) by log[CDDP] vs. normalized response–variable slope. Statistical analysis was carried out by one-way ANOVA followed by Dunnett’s Multiple Comparison test (compare the mean of each column with the mean of a control column) or Turkey’s Multiple Comparison test (compare the mean of each column with the mean of every other column). All statistical analysis was carried out using PRISM Software (Version 6.0; GraphPad, San Diego, CA, USA). *p* values *p* < 0.05, 0.01 and 0.001 were considered to be significant.

## 3. Results

### 3.1. Resv Induces Sensitivity to CDDP in MCF-7_R_ Cells

**Figure 1:**
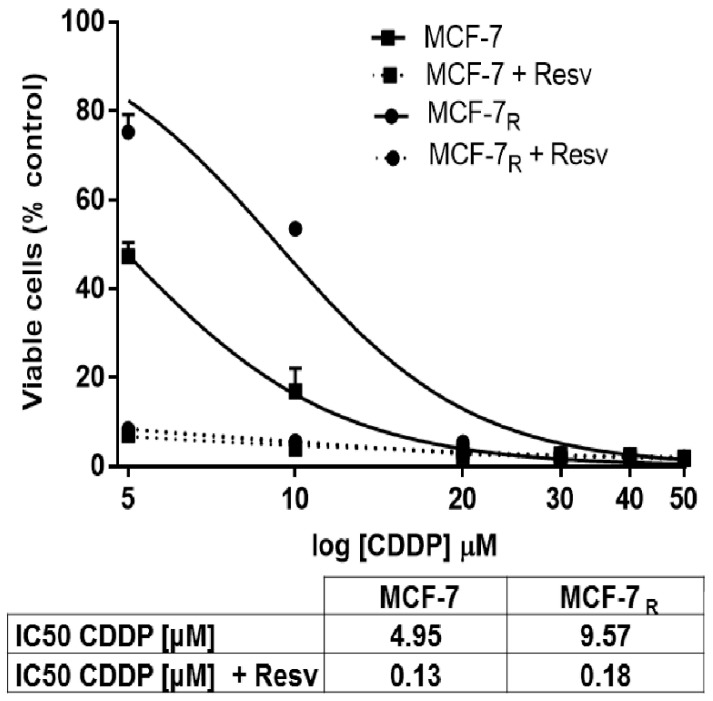
Resveratrol (Resv) induces sensitivity to cisplatin (CDDP) in MCF-7_R_ cells. MCF-7 and MCF-7_R_ cells were treated with different concentrations of CDDP (5, 10, 20, 30, 40, and 50 μM) with or without Resv (100 μM) for 48 h. Cell viability was tested by 3-(4,5-dimethylthiazol-2-yl)-2,5-diphenyltetrazolium bromide (MTT) assay. Each data point is the mean of four independent experiments ± SD. The IC_50_ values for CDDP were calculated and shown in the box.

To determine the effect of Resv in inducing chemosensitivity to MCF-7 and the CDDP-resistant cell line variant (MCF-7_R_); both cells were treated with different CDDP concentrations (5, 10, 20, 30, 40, 50 μM) with or without Resv (100 μM) for 48 h. As shown in Figure 1, we found that the IC_50_ of CDDP was decreased by Resv in both cell lines; in MCF-7 cells the IC_50_ for CDDP was reduced by ~38-fold, from 4.95 μM to 0.13 μM. On the other hand, in MCF-7_R_ cells the IC_50_ of CDDP was decreased by ~53-fold, from 9.57 μM to 0.18 μM. These results suggest that Resv significantly reduced the concentration necessary of CDDP to reach the IC_50_ in both MCF-7 and MCF-7_R_ cells and increases the sensibility to CDDP.

### 3.2. Down-Regulation of p53 Expression and Inhibition of the p53 Protein Activity Enhances Resistance to CDDP and Resv in MCF-7 and MCF-7_R_ Cells

To evaluate the role of p53 in CDDP resistance, MCF-7 and MCF-7_R_ cells were transfected with shRNA targeting p53 (p53-shRNA) or control (Ctrl-shRNA). Stably transfected cells were treated with CDDP (6 μM; 48 h) to stimulate p53 expression. In the presence of p53-shRNA, p53 induction in CDDP treated MCF-7_R_ cells decreased to 40.5% ± 2.3%, when compared to control transfected MCF-7_R_ cells (Supplementary Figure S1A,B, lane 3, *** *p* < 0.001), and the inhibition of p53 induction was higher for MCF-7 p53-shRNA cells (19.7% ± 1.18%), compared to control transfected MCF-7 cells (Supplementary Figure S1A,B lane 5, *** *p* < 0.001).

**Figure 2:**
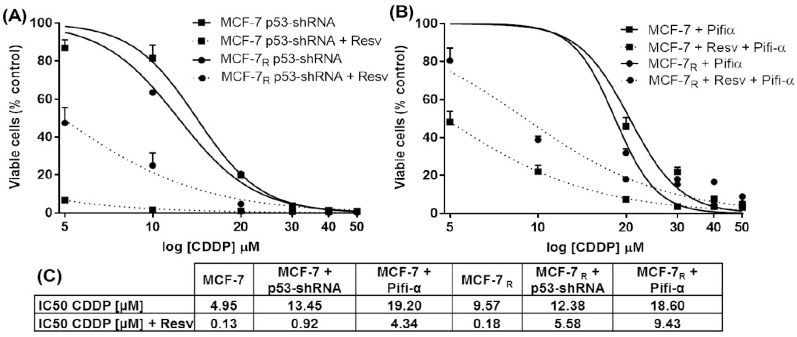
Down-regulation of p53 expression and inhibition of the p53 protein activity enhances resistance to CDDP and Resv in MCF-7 and MCF-7_R_ cells. (**A**) p53-shRNA transfected cells and (**B**) MCF-7 and MCF-7_R_ cells were pretreated with 10 μM of pifithrin-α (Pifi-α) for 24 h; both were treated for 48 h with indicated CDDP concentrations with or without Resv (100 μM). Cell viability was tested by MTT assay. Each data point is the mean of three independent experiments ± SD. (**C**) A summary of the IC_50_ values for CDDP that were calculated by nonlinear regression (curve fit) by log[CDDP] vs. normalized response–variable slope.

To analyze the effect of p53 down-regulation on CDDP and CDDP + Resv treatments, MCF-7 and MCF-7_R_ cells containing p53-shRNA were treated for 48 h with different CDDP concentrations (5, 10, 20, 30, 40, 50 μM) with or without Resv (100 μM). We found an increase in the IC_50_ of CDDP in both treatments and in both cell lines. For MCF-7 p53-shRNA the IC_50_ = 13.45 μM (CDDP) increased ~3-fold; and IC_50_ = 0.92 μM (CDDP + Resv) increased ~7-fold, compared with non-transfected MCF-7 cells (Figure 2A,C). On the other hand, in MCF-7_R_ p53-shRNA the IC_50_ = 12.38 μM (CDDP) increased ~1.3-fold; while IC_50_ = 5.58 μM (CDDP + Resv) increased ~31-fold, compared with non-transfected MCF-7_R_ cells (Figure 2A,C). Interestingly, the increase of IC_50_ for both cell lines was more significant when the CDDP + Resv treatment was used, suggesting that p53 expression plays a more important role in this treatment. Unexpectedly, we found a decrease in the IC_50_ for CDDP of the MCF-7 Ctrl-shRNA and MCF-7_R_ Ctrl-shRNA cells in both treatments (Supplementary Figure S2), compared with non-transfected cells (Figure 1).

To examine the role of p53 transactivation activity in CDDP resistance, MCF-7 and MCF-7_R_ cells were cultured in the presence of pifithrin-α (Pifi-α), an inhibitor of the p53 gene transcription activity. The cells were pretreated for 24 h with 10 μM of Pifi-α and then treated with different CDDP concentrations (5, 10, 20, 30, 40, 50 μM) with or without Resv (100 μM) for 48 h. As shown in Figure 2B,C, a significant increase in the IC_50_ of CDDP was observed in both treatments and in both cell lines. In MCF-7 cells the IC_50_ of CDDP was 19.20 μM (~4-fold increased), and 4.34 μM (CDDP + Resv, ~33-fold increased) compared with MCF-7 cells without pifithrin-α (Figure 2B,C). On the other hand, in MCF-7_R_ cells the IC_50_ of CDDP was 18.60 μM (~2-fold increased), and 9.43 μM (CDDP + Resv, ~52-fold increased), compared with MCF-7_R_ cells without pifithrin-α (Figure 2B,C). Indeed, pifithrin-α enhanced CDDP and CDDP + Resv resistance, probably because inhibition of p53 transactivation activity was more efficient than completely down-regulating p53 expression. Taken together, these results demonstrate that down-regulation of p53 expression or inhibition of p53-dependent gene transcription enhanced chemoresistance to CDDP in MCF-7 and MCF-7_R_ cells under both treatments, suggesting a key role of p53 in overcoming the chemoresistance of MCF-7_R_ cells.

### 3.3. Resv Induces S20 Phosphorylation and Attenuates Phosphorylation of p53 in S15 and S46 in CDDP-Treated MCF-7_R_ Cells

**Figure 3:**
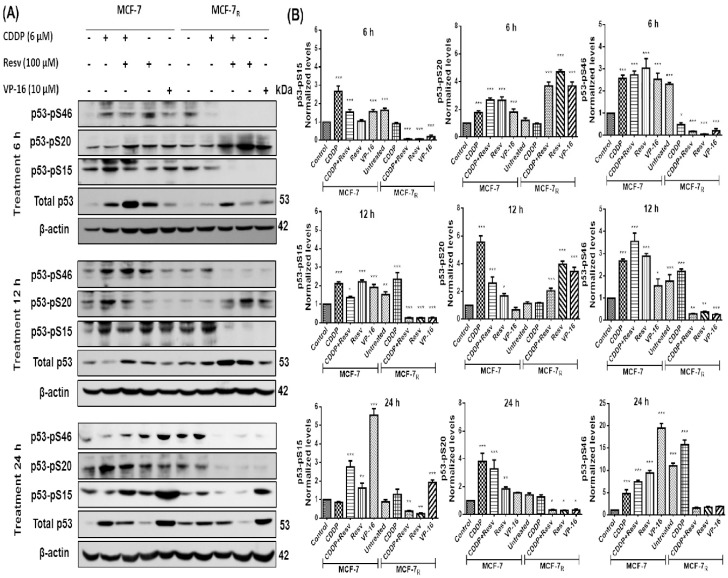
Resv induces serine 20 (S20) phosphorylation and attenuates phosphorylation of p53 in serine 15 (S15) and serine 46 (S46) in CDDP-treated MCF-7_R_ cells. (**A**) MCF-7 and MCF-7_R_ cells were treated with DMSO-ethanol vehicle as control or CDDP (6 μM) with or without Resv (100 μM) for 6, 12, and 24 h. Total and phospho-p53 contents were assessed by Western blot using antibodies directed against total p53 (DO-1) or against specific phosphorylated residues on p53, as indicated. VP-16 (10 μM) treated cells was used as positive control of p53 phosphorylation. (**B**) Densitometric analysis of phospho-p53 after β-actin normalization. One-way ANOVA followed by Dunnett’s Multiple Comparison test were used to compare untreated MCF-7 cells (used as control group) with all the other groups of data at each time point. Results are presented as mean of three independent experiments ± SD. * *p* < 0.05; ** *p* < 0.01; *** *p* < 0.001.

We next evaluate the hypothesis that phosphorylation of p53, which is required for p53-mediated apoptosis, is reduced in response to CDDP [24] in chemoresistant cells, and that Resv activates p53-mediated apoptosis through restoring phosphorylation of p53 in S15 (p53–pS15), S20 (p53–pS20) and S46 (p53–pS46) to chemosensitize MCF-7_R_ cells. We treated MCF-7 and MCF-7_R_ cells with CDDP (6 μM) with or without Resv (100 μM) or Resv alone (100 μM) for 6, 12, and 24 h to analyze p53 phosphorylation status in S15, S20, and S46. In Figure 3A,B, we found that in MCF-7 cells, p53–pS15 phosphorylation after CDDP had its highest peak at 6 h and then gradually diminished (but not completely) at 12 and 24 h; however, for Resv and CDDP + Resv, p53–pS15 phosphorylation was maintained at 6 to 12 h and has highest peak at 24 h. p53–pS20 phosphorylation in CDDP started at 6 h although the highest point was at 12 h. Resv induced S20 phosphorylation at 6 h and diminished at 12 h, with a little increase at 24 h. CDDP + Resv induced a similar behavior than Resv with a moderate rise at 24 h. p53–pS46 phosphorylation was very similar for the three treatments being induced at 6 h and having its highest peak at 24 h. However, in MCF-7_R_ cells, contrary to what we hypothesized, CDDP showed activation of S15 and S46, although it was delayed until 12 h and 24 h, respectively. On the other hand, phosphorylated p53–pS20 was not increased by CDDP treatment as compared with the control (without treatment). Interestingly, CDDP + Resv and Resv treatments showed a converse pattern of p53 phosphorylation by CDDP, phosphorylating S20 at 6 h and 12 h and inhibiting S15 and S46 phosphorylation, suggesting that phosphorylation at S20 is an important event for CDDP resistance and Resv restoration of sensibility. We used VP-16 treatment as positive phosphorylation control for MCF-7-sensitive cells. Interestingly, when used VP-16 treatment in MCF-7_R_ cells we found the same effect as in the treatment with Resv, suggesting the possibility that both have a similar signaling pathway to induce p53 phosphorylation at S20.

### 3.4. Early Phosphorylation of p53 in S20 Induced by Resv Is Sufficient to Activate p53-Dependent Gene Transcription in MCF-7_R_ Cells

**Figure 4:**
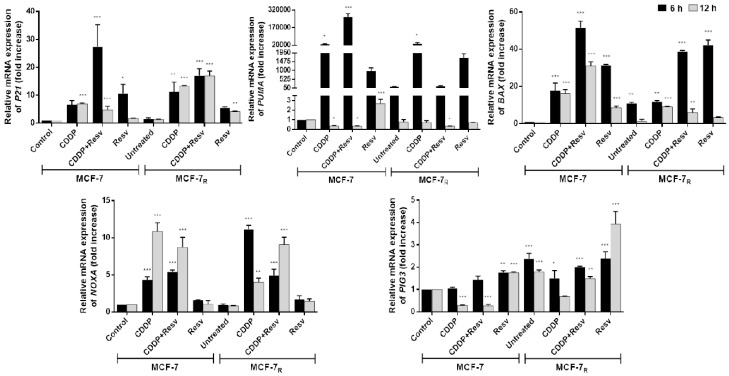
Early phosphorylation of p53 in S20 induced by Resv is sufficient to activate p53-dependent gene transcription in MCF-7_R_ cells. Total RNA extracted from cells treated with DMSO-ethanol vehicle as control or CDDP (6 μM) with or without Resv (100 μM) for 6 and 12 h was assessed for expression levels of *P21*, *BAX*, *NOXA*, *PUMA* and *PIG3* by RT-PCR. The mRNA level of genes was normalized to the *B2M* housekeeping gene. One-way ANOVA followed by Dunnett’s Multiple Comparison test were used to compare untreated MCF-7 cells (used as control group) with all the other groups of data at each time point. Results are presented as mean of three independent experiments ± SD. * *p* < 0.05; ** *p* < 0.01; *** *p* < 0.001.

Stabilized p53 transactivates its target genes promoting cell cycle arrest (e.g., *P21*), DNA repair [9], and apoptosis under severe DNA damage (*PUMA, NOXA, BAX* and *PIG3*) [17,18,19]. We observed that the only phosphorylation of p53 in MCF-7_R_ induced by Resv was at S20, so we treated MCF-7 and MCF-7_R_ cells with CDDP (6 μM) with or without Resv (100 μM) or Resv alone (100 μM) for 6 and 12 h to evaluate whether this phosphorylation is sufficient to activate p53-dependent gene transcription in MCF-7_R_ cells. RT-qPCR was used to determine the mRNA level of the mentioned genes. As shown in Figure 4, expression of all genes was triggered at 6 h. *P21* and *PUMA* genes were highly up-regulated by all conditions of treatment (CDDP with or without Resv or Resv alone). *NOXA* was elevated by CDDP and CDDP + Resv, although activation by the combination was lower and the maximum peak was at 12 h. Perhaps in combination Resv hinders CDDP activation, since Resv alone does not induce *NOXA*. On the other hand, *PIG3* barely responded to Resv alone (nearly 4-fold after Resv treatment in MCF-7_R_ cells) suggesting null participation of this gene. Unexpectedly, there does not seem to be a synergy between the treatments, since activation of all genes in the combination treatment always was lower than in CDDP or Resv alone, suggesting that just one of them is responsible for the activation of a particular gene. Interestingly, *BAX*, one of the main apoptotic effectors, is only activated by Resv, indicating this could be a key event for the induction of apoptosis in MCF-7_R_ cells.

Taken together, these data suggest that early phosphorylation of p53 in S20 induced by Resv in MCF-7_R_ cells is sufficient to activate p53-dependent gene transcription of selected genes and does not require phosphorylation of p53 in S15 and S46.

### 3.5. Resv Overcome CDDP-Resistance and Induces Apoptosis in MCF-7_R_ Cells

**Figure 5:**
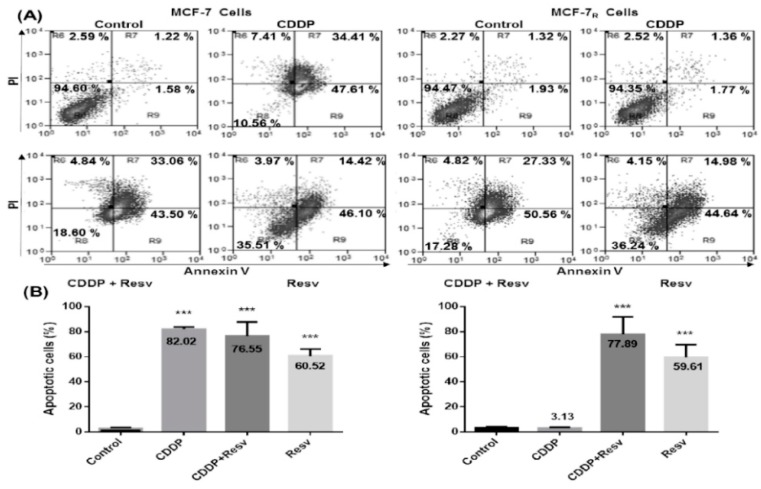
Resv overcome CDDP-resistance and induces apoptosis in MCF-7_R_ cells. (**A**) MCF-7 and MCF-7_R_ cells were treated with a DMSO–ethanol vehicle as control or CDDP (6 μM) with or without Resv (100 μM) for 48 h and were double-stained with Annexin V and propidium iodide (PI) followed by flow cytometry analysis to determine apoptotic cells. The viable cells are located in the lower left quadrant (double negative with Annexin V–/PI–). Apoptotic cells (Annexin V+/PI–) appear in the lower right (early apoptosis) and upper right (late apoptosis) quadrant of data plots. Data are presented as percentage of the cell population. (**B**) The combined results of three independent cytometry analyses depicting the mean levels of total apoptotic cells are shown. Results are presented as the means ± SD. *** *p* < 0.001 by one-way ANOVA followed by Dunnett’s Multiple Comparison test.

We evaluated the induction of apoptosis triggered by Resv by flow cytometry using Annexin V/PI in MCF-7 and MCF-7_R_ cells treated with CDDP (6 μM) with or without Resv (100 μM) for 48 h. Figure 5A shows the percentage of total apoptosis (early and late apoptosis) for MCF-7 cells (left panel) with CDDP treatment was 82.02% ± 1.79%, for CDDP + Resv it was 76.55% ± 11.16%, and for Resv alone it was 60.52% ± 5.57% (Figure 5B, *p <* 0.001, left graph). As expected, MCF-7_R_ cells (right panel) treated with CDDP did not show apoptosis; however, with CDDP + Resv treatment showed 77.89% ± 13.80% and Resv alone 59.61% ± 10.16% total apoptotic cells, similar to their chemosensitive counterpart (Figure 5B, *p <* 0.001, right graph). These data suggest that Resv with or without CDDP induces apoptosis in chemoresistant MCF-7_R_ cells.

### 3.6. Early Phosphorylation of p53 in S20 Induced by Resv Is Necessary for p53-Stability in MCF-7_R_ Cells

**Figure 6:**
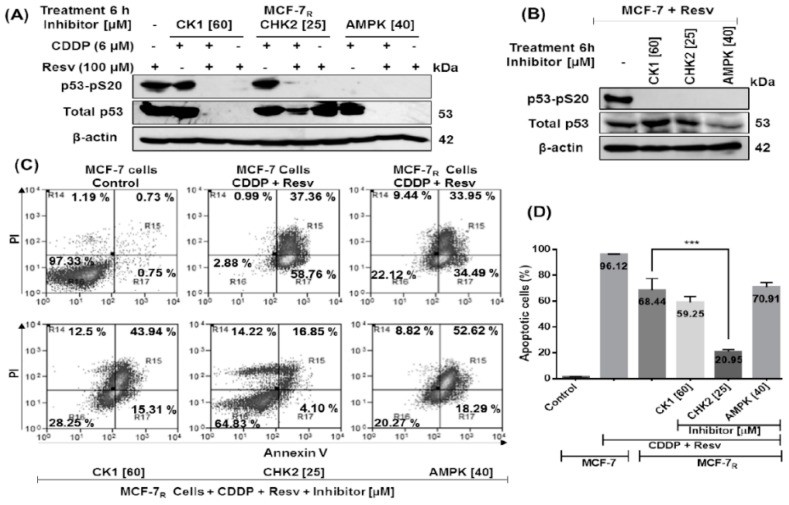
Early phosphorylation of p53 in S20 induced by Resv is necessary for p53-stability in MCF-7_R_ cells. (**A**) MCF-7_R_ cells were treated with CDDP (6 μM) with or without Resv (100 μM) and (**B**) MCF-7 cells were treated with Resv (100 μM); both cell cultures were treated for 6 h with specific p53-pS20 site kinase inhibitors: CK1 (60 μM), CHK2 (25 μM) or AMPK (40 μM). Total and phospho-p53 contents are assessed by Western blot using antibodies directed against total p53 (DO-1) or against the specific phosphorylated residue on S20, as indicated. (**C**) MCF-7 and MCF-7_R_ cells were treated with a DMSO–ethanol vehicle as control or CDDP (6 μM) with resveratrol (100 μM) and cultured in combination with CK1 (60 μM), CHK2 (25 μM) or AMPK (40 μM) inhibitors for 48 h and were double-stained with Annexin V and propidium iodide (PI) followed by flow cytometry analysis to determine apoptotic cells. The viable cells are located in the lower left quadrant (double negative with Annexin V–/PI–). Apoptotic cells (Annexin V+/PI–) appear in the lower right (early apoptosis) and upper right (late apoptosis) quadrant of data plots. Data are presented as a percentage of the cell population. (**D**) The combined results of three independent cytometry analyses depicting the mean levels of total apoptotic cells are shown. Results are presented as the means ± SD. *** *p* < 0.001 by one-way ANOVA followed by Turkey’s Multiple Comparison test.

It has been reported that CK1, CHK2, and AMPK can induce p53-pS20 phosphorylation in response to various types of stress such as CK1 in virus infection (DNA virus HHV-6B) [25], ionizing radiation for CHK2 [26], and metabolic stress for AMPK [27]. To elucidate which activation signal is induced by Resv to phosphorylate S20, we treated MCF-7_R_ cells with CDDP (6 μM) with or without Resv (100 μM) in the presence of specific p53–pS20 kinase inhibitors: CK1 inhibitor D4476 (60 μM), CHK2 inhibitor (25 μM), or AMPK inhibitor compound C (40 μM) during 6 h. As shown in Figure 6A, inhibition of S20 phosphorylation by CK1 and CHK2 inhibitors only take place in Resv treatment; while inhibition of AMPK impeded S20 phosphorylation in both CDDP and Resv treatments. We found that in CDDP treated cells only the AMPK inhibitor blocks S20 phosphorylation but unexpectedly all three inhibitors block p53-pS20 phosphorylation in Resv-treated cells. Furthermore, in the CDDP treatment with AMPK inhibitor the p53 stability was unaffected given that in MCF-7_R_ cells treated with CDDP, p53 was also phosphorylated on S15 and S46 (see Figure 3A and Figure 6A). However, Resv treatment inhibits p53–pS15 and p53–pS46 phosphorylation in MCF-7_R_ cells (see Figure 3A), consequently loss of S20 phosphorylation by AMPK and CK1 inhibitors resulted in a complete impairment of p53 stability (Figure 6A). On the other hand, with the CHK2 inhibitor, p53 stability was not affected, suggesting that in the presence of Resv another post-translational modification in p53 is involved in an attenuation of the effect of p53–pS20 loss.

In order to compare the effect of the inhibitors with their chemosensitive counterpart, we also treated MCF-7 cells with Resv (100 μM) and with specific p53–pS20 site kinase inhibitors for 6 h. As shown in Figure 6B, CK1, CHK2 and AMPK inhibitors suppress p53–pS20 phosphorylation without degradation of p53 since p53–pS15 and p53–S46 phosphorylations are induced by Resv (see Figure 3A and Figure 6A). All together these data suggest that in MCF-7_R_ cells the early phosphorylation of p53 in S20 induced by Resv is sufficient for p53 stabilization and their transactivation function and that its inhibition induces p53 degradation compared with their chemosensitive counterpart where p53 is still stable after the inhibition of p53–pS20, probably because it contains phosphorylation in S15 and S46 induced by Resv.

To investigate the effect that the inhibitors had in p53-induced apoptosis, we treated MCF-7_R_ cells with CDDP (6 μM) + Resv (100 μM) and with specific p53–pS20 kinase inhibitors for 48 h and evaluated the induction of apoptosis with Annexin V/PI and flow cytometry. As shown in Figure 6C, the MCF-7_R_ cells treated with CK1 and AMPK inhibitors (degraded p53) had 59.25% ± 4.27 and 70.91% ± 3.43% total apoptotic cells, respectively, suggesting a p53-independent apoptosis. On the other hand, the MCF-7_R_ cells in the presence of CHK2 inhibitor (low p53 level) showed a significant reduction of apoptotic cells with 20.95% ± 1.43% vs. 68.44% ± 8.94% of apoptotic cells without inhibitor (Figure 6D, *** *p <* 0.001), suggesting that the presence of a non-functional p53 form in MCF-7_R_ cells (without phosphorylation in S15, S20 and S46) can hamper the induction of apoptosis.

### 3.7. Resv Promotes Early Dephosphorylation of ATM, Inhibition of BCL-2, and Upregulation of BAX

**Figure 7:**
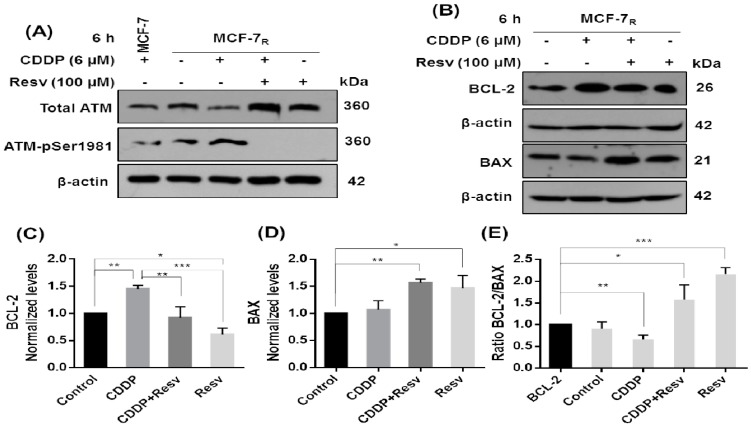
Resv promotes early dephosphorylation of ATM, inhibition of BCL-2, and upregulation of BAX. MCF-7_R_ cells were treated with CDDP (6 μM) with or without Resv (100 μM) for 6 h. (**A**) Total and phospho-ATM and (**B**) BCL-2 and BAX proteins were assessed by Western blot using antibodies directed against total ATM, a specific phosphorylated residue on S1981 of ATM, BCL-2, or BAX. MCF-7 cells with CDPP were used as positive control of ATM phosphorylation. (**C**) Densitometric analysis of BCL-2 and (**D**) BAX after β-actin normalization. (**E**) Ratio between BCL-2/BAX by *t* test. All results are presented as mean of three independent experiments ± SD. * *p* < 0.05; ** *p* < 0.01; *** *p* < 0.001 by one-way ANOVA followed by Turkey’s or Dunnett’s Multiple Comparison test.

It has been reported that S15 of p53 is phosphorylated by activated ATM (S1981-phosphorylated ATM) at an earlier inductive phase after DNA damage [14,15]. S46 is also sequentially phosphorylated by ATM [15]; supporting these observations, we found that the treatment of MCF-7_R_ cells with Resv with or without CDDP for 6 h promotes early deactivation of ATM by dephosphorylation in S1981 regardless of the total ATM level (Figure 7A), so it is possible that the decrease in p53 phosphorylation in S15 and S46 MCF-7_R_ cells (see Figure 3A) could be due to dephosphorylation of ATM by Resv. On the other hand, to investigate the blockade of apoptosis in MCF-7_R_ cells treated with CDDP, we analyzed the ratio of anti-apoptotic BCL-2 and proapoptotic BAX proteins, finding that BCL-2 was elevated while BAX was decreased after 6 h treatment with CDDP. On the other hand, in cells treated with CDDP + Resv or only Resv, BCL-2 protein expression was decreased while at the same time BAX was increased (Figure 7B–E). This result suggests that elevated BCL-2 in CDDP treatment blocked apoptosis and that Resv partly induces apoptosis by changing the ratio between BCL-2 and BAX proteins.

## 4. Discussion and Conclusions

CDDP is one of the most widely used anticancer drugs in the treatment of various types of cancer, including human breast cancer [28], but its use commonly results in adverse effects and toxicities affecting healthy systems, with resistance a major cause of treatment failure [1,29]. Therefore, it is of interest to continue searching for effective chemosensitizers. Resv is known to be an anticancer and protective agent which has the potential for preventing CDDP-related toxicity; it can sensitize chemoresistant cells by overcoming mechanisms of chemoresistance, including the upregulation of p53 [7,9,10]. Considering the chemosensitizer capacity of Resv, we developed a CDDP-resistant MCF-7_R_ cell line variant employing only 6 μM of CDDP because at higher concentrations (>6.5 μM) the cells died or formed clusters into the medium that prevented the formation of cell monolayers; a similar effect was previously described in other CDDP-resistant cancer cells [30,31]. Our results showed that Resv induces CDDP sensitivity, decreasing the IC_50_ of CDDP in MCF-7 and our MCF-7_R_ cells.

The contribution of p53 to chemosensitivity and chemoresistance remains partly unclear. It has been reported that acquisition of resistance to chemotherapeutic drugs including CDDP also occurs in cancer cells expressing p53 wt. One mechanism proposed to explain this phenomenon is that this p53 protein becomes inactive. p53 could be activated by phosphorylation in response to various cell stress signals, protecting p53 from MDM2-mediated ubiquitination and proteasomal degradation. Phosphorylation of p53 in S15 and S20 is required to perform DNA repair [14,15], and under severe DNA damage, S46 is also sequentially phosphorylated for p53-induced apoptosis [13,16]. We think that resistance of MCF-7 cells to CDDP could be related to the lack of phosphorylation in these specific sites of the p53 protein as was described previously in CDDP-resistant ovarian cancer cells [24]. We treated MCF-7 and MCF-7_R_ cells with 6 μM of CDDP (maximal concentration for the survival of chemoresistant cells) to compare the effect in both cell lines. Our data showed that the inhibition of p53 expression (p53 shRNA) or its transactivation activity (pifithrin-α) enhances the resistance of both cell lines to CDDP and CDDP + Resv, suggesting the active participation of p53 after drug treatment. This effect was also observed in other reports that show that the downregulation of p53 enhances CDDP resistance [32,33] and importantly, Resv also has been reported to induce apoptosis through a p53-dependent pathway [9,10]. CDDP treatment induced p53 phosphorylation of S15, S20, and S46 in MCF-7 cells; in MCF-7_R_ cells S15 and S46, also appeared to be constitutively phosphorylated even without treatment, but these cells survive. Interestingly, S20 phosphorylation was inhibited in CDDP-treated MCF-7_R_ cells while at the same time was strongly enhanced in CDDP + Resv and Resv treatments, suggesting that S20 phosphorylation could be key for p53 to activate target genes, specifically *BAX*, to overcome CDDP resistance in MCF-7_R_ cells. Furthermore, the importance of this site is highlighted by the fact that the treatment with CDDP in combination with Resv or Resv alone attenuated p53 phosphorylation at S15 and S46 but promoted apoptosis. However, we do not discard the possible phosphorylation of p53 in other sites that collaborate with S20 to induce apoptosis. Interestingly, we found the same effect observed for Resv in chemoresistant cells treated with VP-16, suggesting that both compounds have a similar signaling pathway to induce p53 phosphorylation in S20. Regarding the inhibition of phosphorylation in S15 and S46 in MCF-7_R_ cells, it is most probably related to the loss of ATM activation in CDDP + Resv and Resv treatments, consistent with our results (Figure 7) and reports that describe that S15 of p53 is phosphorylated by activated ATM at an early phase after DNA damage [14,15], and then S46 is sequentially phosphorylated by ATM [20]. Furthermore, since ATM activity is a key regulator of DNA damage response that is related to genotoxic resistance, the inhibition of ATM activity [34,35] could also contribute to the chemosensitivity of MCF-7_R_ cells. Previously, it was reported that Resv induced phosphorylation of p53 in S15 and S20 in MCF-7 cells [20,21], but to our knowledge this is the first time that it has been shown that Resv also induces phosphorylation in S46. Our results are consistent with reports in MCF-7 cells and in several chemosensitive and chemoresistant cancers indicating that Resv increases p53-dependent transcriptional activity including increase of mRNA levels of *BAX*, *BAK,* and *PUMA* [36,37].

In order to elucidate which kinase pathway is responsible for p53–pS20 activation in MCF-7_R_ cells, we used three known specific inhibitors of kinases that phosphorylate p53 in S20 which include the DNA damage pathway (CHK2 inhibitor), oncogene activation (CK1 inhibitor), and metabolic stress (AMPK inhibitor). We observed that the low continuous phosphorylation of S20 in CDDP treated MCF-7_R_ cells is induced by AMPK since it was sensitive to the AMPK inhibitor. Activation of AMPK by CDDP has been previously reported, and it was related to apoptosis inhibition and acquired resistance [38,39]. It is very interesting that the kinase responsible for S20 phosphorylation by CDDP is the same that could be responsible for apoptosis inhibition. Surprisingly, when we used the three inhibitors in CDDP + Resv and Resv treated MCF-7_R_ cells, all of them blocked S20 phosphorylation, suggesting that Resv activates the three kinases to phosphorylate p53. At this point we cannot explain the codependence of the three kinases to phosphorylate S20 but it is possible that Resv activates the three kinases to assure or maintain phosphorylation for a longer time. Under this scenario, we think that there could be a fluid dynamic between the three enzymes for the interaction in the docking site for S20 and the hampering of any of the enzymes could block the site for the other two. There is also the possibility of an unknown cross-talk between them or that the interaction of the three kinases in the docking sites of Box-V domain of p53 was also important for allowing S20 phosphorylation. Nevertheless, this interesting result should be analyzed further in future works. Additionally, the inhibition of S20 phosphorylation by CK1 and AMPK kinase inhibitors in MCF-7_R_ results in loss of p53 stability, while the inhibition of CHK2 conserves some of the p53 total protein expression in the presence of Resv, suggesting that other phosphorylation sites for CHK2 along the p53 protein could be essential to maintain p53 stability. Although the three kinases were necessary in phosphorylating p53–pS20, we performed apoptosis assays in the presence of each of the three inhibitors to elucidate if one of the kinases is key or more important for the activation of apoptosis. Unfortunately, complete loss of p53 stability with CK1 and AMPK inhibitors produced an elevated induction of apoptosis, masking the object of the experiment. Although the result was unsought, there have been some works describing the same phenomenon in MCF-7 cells. For example, in a study in MCF-7 cells, disruption of p53 with a plasmid expressing the E6 oncoprotein sensitizes them to CDDP [40]. In the same manner, Mendez and Lupu silenced p53 to elucidate if the apoptosis induced in MCF-7 cells by the inhibition of FASN was through the p53 pathway; unexpectedly, they found an elevation of 300% in apoptosis [41]. Also, specific down-regulation of p53 showed an increase in apoptosis via SMAD4 [42]. Finally, using a RNAi for p53 also sensitized MCF-7 cells to apoptosis induced by ceramide [43]. These observations could partially explain our results since the treatments we used were CDDP and Resv, which are known to induce apoptosis also by ceramide induction. However, another interesting observation was that with the CHK2 inhibitor some of the total but probably inactive p53 protein was conserved; the induction of apoptosis was strongly diminished, suggesting that inactive p53 protein not only diminished the induction of apoptosis but also blocked it. Finally, we also observed another important difference in BCL2–*BAX* balance between CDDP and CDDP + Resv treated MCF-7_R_ cells. First, RT-qPCR results show that pro-apoptotic *BAX* gene expression was highly elevated in CDDP + Resv and Resv treatments, while in the CDDP treatment it was slightly decreased. On the other hand, anti-apoptotic BCL-2 protein was elevated in CDDP treatment, while in CDDP + Resv and Resv treatments the BCL-2 protein expression was diminished. As previously reported, the balance between BCL-2 and BAX is a key regulatory element [44] and could be an additional mechanism explaining the induction of apoptosis in MCF-7_R_ cells in the presence of Resv or CDDP + Resv. Our results suggest a new model of chemosensitization by Resv in MCF-7_R_ cells, involving phosphorylation in p53–pS20. This model is in accord with our previous observation of Resv sensitizing MCF-7 cells by downregulation of RAD51 since p53 could repress RAD51 mRNA and protein expression [23,45].

**Figure 8:**
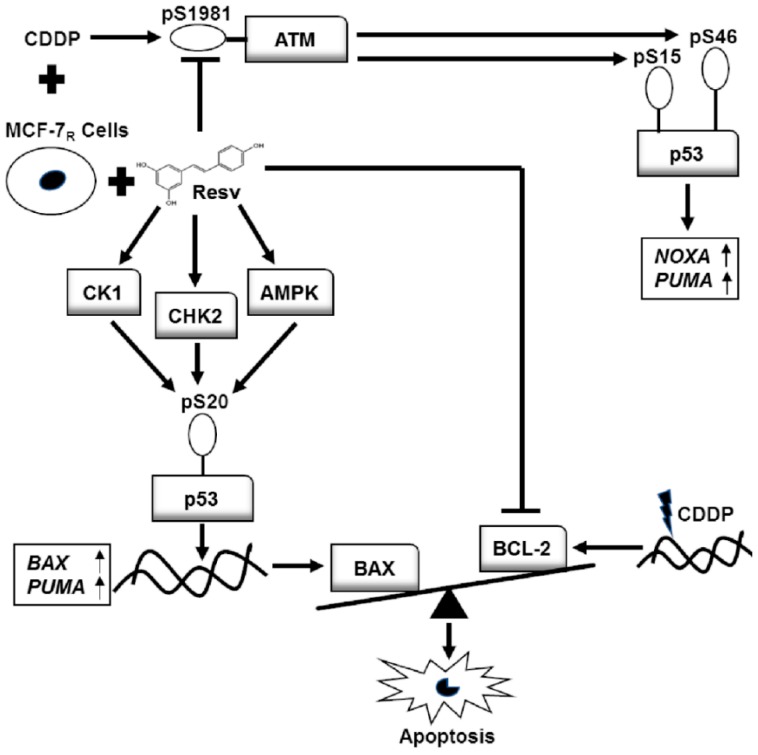
In the MCF-7 resistant cell variant (MCF-7_R_), Resv attenuates phosphorylation in S15 and S46 of p53 by dephosphorylation and deactivation of ATM. However, it activates kinases CK1, CHK2, and AMPK to induce phosphorylation of p53 in S20 (which is required to activate p53 in order to upregulate *BAX* and *PUMA* genes) and modifies the ratio between BCL-2/BAX expression. The BAX protein was increased while BCL-2 protein was decreased, restoring apoptosis and overcoming chemoresistance. On the other hand, the overexpression of BCL-2 in MCF-7_R_ cells after CDDP treatment maintains the chemoresistance and blocks apoptosis despite the phosphorylation of p53 in S15 and S46 and the upregulation of *NOXA* and *PUMA*.

Our results show that Resv reduces the IC_50_ of CDDP necessary to induce apoptosis in chemosensitive and in CDDP-resistant MCF-7 cell line variant, increasing the capability to arrest, delay or reverse carcinogenesis in an adjuvant CDDP therapy. This study provides evidence on the role of p53 for a potential CDDP acquired resistance model and the molecular mechanism of Resv to chemosensitize resistant breast cancer cells to CDDP. We demonstrated for a resistant cell line variant that down-regulation of p53 and inhibition of p53-dependent gene transcription enhanced chemoresistance to CDDP in chemosensitive and chemoresistant cells, suggesting that the chemosensitization to CDDP by Resv is mainly p53-dependent. Moreover, in chemoresistant cells Resv induces early phosphorylation of p53 in S20 and attenuates CDDP-induced p53 phosphorylation in S15 and S46 residues, probably through dephosphorylation and deactivation of ATM. This phosphorylation in p53–pS20 is sufficient to activate p53-dependent gene transcription including *PUMA* and *BAX* genes restoring apoptosis in MCF-7_R_ cells. Resv activates different kinases, such as CK1, CHK2, and AMPK to induce phosphorylation of p53 in S20, suggesting a novel mechanism of p53 activation and chemosensitization to CDDP. At the same time, Resv downregulates BCL-2 expression, a key player in apoptosis inhibition. On the other hand, CDDP induces p53 phosphorylation in chemoresistant cells but the apoptosis is probably blocked downstream at least in part by the up-regulation of BCL-2 protein despite the up-regulation of *PUMA* and *NOXA* (see model in Figure 8). A more thorough understanding of the molecular mechanism underlying this particular chemoresistance and the chemosensitization by Resv in this resistant cell variant may ultimately help for improvement in the treatment of human breast cancer.

## Supplementary Materials

The following are available online at http://www.mdpi.com/2072-6643/10/9/1148/s1, Figure S1: Down-regulation of p53 in MCF-7 and MCF-7_R_ cells by shRNA; Figure S2: Transfected cells with Ctrl-shRNA are sensitive to Resv; Table S1: Primers for RT-qPCR p53 target gene analysis.

## References

[1] Brabec V., Kasparkova J. (2005). Modifications of DNA by platinum complexes. Relation to resistance of tumors to platinum antitumor drugs. Drug Resist. Updat..

[2] Kaur G., Verma N. (2015). Nature curing cancer—Review on structural modification studies with natural active compounds having anti-tumor efficiency. Biotechnol. Rep. (Amst.).

[3] Fulda S. (2010). Resveratrol and derivatives for the prevention and treatment of cancer. Drug Discov. Today.

[4] Fabris S., Momo F., Ravagnan G., Stevanato R. (2008). Antioxidant properties of resveratrol and piceid on lipid peroxidation in micelles and monolamellar liposomes. Biophys. Chem..

[5] Caruso F., Tanski J., Villegas-Estrada A., Rossi M. (2004). Structural basis for antioxidant activity of trans-resveratrol: Ab initio calculations and crystal and molecular structure. J. Agric. Food Chem..

[6] Aggarwal B.B., Bhardwaj A., Aggarwal R.S., Seeram N.P., Shishodia S., Takada Y. (2004). Role of resveratrol in prevention and therapy of cancer: Preclinical and clinical studies. Anticancer Res..

[7] Gupta S.C., Kannappan R., Reuter S., Kim J.H., Aggarwal B.B. (2011). Chemosensitization of tumors by resveratrol. Ann. N. Y. Acad. Sci..

[8] Miyashita T., Krajewski S., Krajewska M., Wang H.G., Lin H.K., Liebermann D.A., Hoffman B., Reed J.C. (1994). Tumor suppressor p53 is a regulator of bcl-2 and bax gene expression in vitro and in vivo. Oncogene.

[9] Ferraz da Costa D.C., Casanova F.A., Quarti J., Malheiros M.S., Sanches D., Dos Santos P.S., Fialho E., Silva J.L. (2012). Transient transfection of a wild-type p53 gene triggers resveratrol-induced apoptosis in cancer cells. PLoS ONE.

[10] Huang C., Ma W.Y., Goranson A., Dong Z. (1999). Resveratrol suppresses cell transformation and induces apoptosis through a p53-dependent pathway. Carcinogenesis.

[11] Gogada R., Prabhu V., Amadori M., Scott R., Hashmi S., Chandra D. (2011). Resveratrol induces p53-independent, x-linked inhibitor of apoptosis protein (xiap)-mediated bax protein oligomerization on mitochondria to initiate cytochrome c release and caspase activation. J. Biol. Chem..

[12] Prabhu V., Srivastava P., Yadav N., Amadori M., Schneider A., Seshadri A., Pitarresi J., Scott R., Zhang H., Koochekpour S. (2013). Resveratrol depletes mitochondrial DNA and inhibition of autophagy enhances resveratrol-induced caspase activation. Mitochondrion.

[13] Taira N., Nihira K., Yamaguchi T., Miki Y., Yoshida K. (2007). Dyrk2 is targeted to the nucleus and controls p53 via ser46 phosphorylation in the apoptotic response to DNA damage. Mol. Cell.

[14] Shieh S.Y., Ikeda M., Taya Y., Prives C. (1997). DNA damage-induced phosphorylation of p53 alleviates inhibition by mdm2. Cell.

[15] Kodama M., Otsubo C., Hirota T., Yokota J., Enari M., Taya Y. (2010). Requirement of ATM for rapid p53 phosphorylation at Ser46 without ser/thr-gln sequences. Mol. Cell. Biol..

[16] D’Orazi G., Cecchinelli B., Bruno T., Manni I., Higashimoto Y., Saito S., Gostissa M., Coen S., Marchetti A., Del Sal G. (2002). Homeodomain-interacting protein kinase-2 phosphorylates p53 at Ser 46 and mediates apoptosis. Nat. Cell Biol..

[17] Villunger A., Michalak E.M., Coultas L., Mullauer F., Bock G., Ausserlechner M.J., Adams J.M., Strasser A. (2003). P53- and drug-induced apoptotic responses mediated by bh3-only proteins puma and noxa. Science.

[18] Kong W., Jiang X., Mercer W.E. (2009). Downregulation of wip-1 phosphatase expression in mcf-7 breast cancer cells enhances doxorubicin-induced apoptosis through p53-mediated transcriptional activation of bax. Cancer Biol. Ther..

[19] Zhang W., Luo J., Chen F., Yang F., Song W., Zhu A., Guan X. (2015). Brca1 regulates pig3-mediated apoptosis in a p53-dependent manner. Oncotarget.

[20] Hsieh T.C., Wong C., John Bennett D., Wu J.M. (2011). Regulation of p53 and cell proliferation by resveratrol and its derivatives in breast cancer cells: An in silico and biochemical approach targeting integrin alphavbeta3. Int. J. Cancer.

[21] Zhang S., Cao H.J., Davis F.B., Tang H.Y., Davis P.J., Lin H.Y. (2004). Oestrogen inhibits resveratrol-induced post-translational modification of p53 and apoptosis in breast cancer cells. Br. J. Cancer.

[22] Leon-Galicia I., Diaz-Chavez J., Garcia-Villa E., Uribe-Figueroa L., Hidalgo-Miranda A., Herrera L.A., Alvarez-Rios E., Garcia-Mena J., Gariglio P. (2013). Resveratrol induces downregulation of DNA repair genes in mcf-7 human breast cancer cells. Eur. J. Cancer Prev..

[23] Leon-Galicia I., Diaz-Chavez J., Albino-Sanchez M.E., Garcia-Villa E., Bermudez-Cruz R., Garcia-Mena J., Herrera L.A., Garcia-Carranca A., Gariglio P. (2018). Resveratrol decreases rad51 expression and sensitizes cisplatinresistant mcf7 breast cancer cells. Oncol. Rep..

[24] Fraser M., Bai T., Tsang B.K. (2008). Akt promotes cisplatin resistance in human ovarian cancer cells through inhibition of p53 phosphorylation and nuclear function. Int. J. Cancer.

[25] MacLaine N.J., Oster B., Bundgaard B., Fraser J.A., Buckner C., Lazo P.A., Meek D.W., Hollsberg P., Hupp T.R. (2008). A central role for ck1 in catalyzing phosphorylation of the p53 transactivation domain at serine 20 after hhv-6b viral infection. J. Biol. Chem..

[26] Craig A., Scott M., Burch L., Smith G., Ball K., Hupp T. (2003). Allosteric effects mediate chk2 phosphorylation of the p53 transactivation domain. EMBO Rep..

[27] Hawley S.A., Boudeau J., Reid J.L., Mustard K.J., Udd L., Makela T.P., Alessi D.R., Hardie D.G. (2003). Complexes between the lkb1 tumor suppressor, strad alpha/beta and mo25 alpha/beta are upstream kinases in the amp-activated protein kinase cascade. J. Biol..

[28] Zhang J., Wang L., Xing Z., Liu D., Sun J., Li X., Zhang Y. (2010). Status of bi- and multi-nuclear platinum anticancer drug development. Anticancer Agents Med. Chem..

[29] Tsang R.Y., Al-Fayea T., Au H.J. (2009). Cisplatin overdose: Toxicities and management. Drug Saf..

[30] Maubant S., Staedel C., Gauduchon P. (2002). Integrins, cell response to anti-tumor agents and chemoresistance. Bull. Cancer.

[31] Nista A., Leonetti C., Bernardini G., Mattioni M., Santoni A. (1997). Functional role of alpha4beta1 and alpha5beta1 integrin fibronectin receptors expressed on adriamycin-resistant mcf-7 human mammary carcinoma cells. Int. J. Cancer.

[32] Nadkarni A., Rajesh P., Ruch R.J., Pittman D.L. (2009). Cisplatin resistance conferred by the rad51d (e233g) genetic variant is dependent upon p53 status in human breast carcinoma cell lines. Mol. Carcinog..

[33] Jiang M., Yi X., Hsu S., Wang C.Y., Dong Z. (2004). Role of p53 in cisplatin-induced tubular cell apoptosis: Dependence on p53 transcriptional activity. Am. J. Physiol. Ren. Physiol..

[34] Weber A.M., Ryan A.J. (2015). ATM and ATR as therapeutic targets in cancer. Pharmacol. Ther..

[35] Luong K.V., Wang L., Roberts B.J., Wahl J.K., Peng A. (2016). Cell fate determination in cisplatin resistance and chemosensitization. Oncotarget.

[36] Sakamoto T., Horiguchi H., Oguma E., Kayama F. (2010). Effects of diverse dietary phytoestrogens on cell growth, cell cycle and apoptosis in estrogen-receptor-positive breast cancer cells. J. Nutr. Biochem..

[37] Shankar S., Chen Q., Siddiqui I., Sarva K., Srivastava R.K. (2007). Sensitization of trail-resistant lncap cells by resveratrol (3, 4′, 5 tri-hydroxystilbene): Molecular mechanisms and therapeutic potential. J. Mol. Signal..

[38] Kim H.S., Hwang J.T., Yun H., Chi S.G., Lee S.J., Kang I., Yoon K.S., Choe W.J., Kim S.S., Ha J. (2008). Inhibition of amp-activated protein kinase sensitizes cancer cells to cisplatin-induced apoptosis via hyper-induction of p53. J. Biol. Chem..

[39] Harhaji-Trajkovic L., Vilimanovich U., Kravic-Stevovic T., Bumbasirevic V., Trajkovic V. (2009). Ampk-mediated autophagy inhibits apoptosis in cisplatin-treated tumour cells. J. Cell. Mol. Med..

[40] Fan S., Smith M.L., Rivet D.J., Duba D., Zhan Q., Kohn K.W., Fornace A.J., O’Connor P.M. (1995). Disruption of p53 function sensitizes breast cancer mcf-7 cells to cisplatin and pentoxifylline. Cancer Res..

[41] Menendez J.A., Lupu R. (2005). RNA interference-mediated silencing of the p53 tumor-suppressor protein drastically increases apoptosis after inhibition of endogenous fatty acid metabolism in breast cancer cells. Int. J. Mol. Med..

[42] Wu B., Li W., Qian C., Zhou Z., Xu W., Wu J. (2012). Down-regulated p53 by sirna increases smad4′s activity in promoting cell apoptosis in mcf-7 cells. Eur. Rev. Med. Pharmacol. Sci..

[43] Struckhoff A.P., Patel B., Beckman B.S. (2010). Inhibition of p53 sensitizes mcf-7 cells to ceramide treatment. Int. J. Oncol..

[44] Delbridge A.R., Grabow S., Strasser A., Vaux D.L. (2016). Thirty years of bcl-2: Translating cell death discoveries into novel cancer therapies. Nat. Rev. Cancer.

[45] Arias-Lopez C., Lazaro-Trueba I., Kerr P., Lord C.J., Dexter T., Iravani M., Ashworth A., Silva A. (2006). P53 modulates homologous recombination by transcriptional regulation of the rad51 gene. EMBO Rep..

