# Supplementary material for: Induction of p53 Phosphorylation at Serine 20 by Resveratrol Is Required to Activate p53 Target Genes, Restoring Apoptosis in MCF-7 Cells Resistant to Cisplatin
Source: Nutrients. 2018 Aug 23;10(9):1148. doi: 10.3390/nu10091148 (PMC6163170; doi:10.3390/nu10091148)
Supplement: Supplementary file 1 [file nutrients-10-01148-s001.zip › Table S1.docx]

**Table S1.** Primers for RT-qPCR p53 target gene analysis.

| Target | Sense primer (5’ to 3’) | Antisense primer (5’ to 3’) |
| --- | --- | --- |
| *P21*  *PUMA*  *BAX*  *PIG3*  *NOXA*  *B2M* | GGAAGACCATGTGGACCTGT  CACCTAATTGGGCTCCATCT  AAAGATGGTCACGGTCCAAC  GGTCACTGGGTAGATTCTGTC  GTGCCCTTGGAAACGGAAGA  ACCTCCATGATGCTGCTTAC | GGCGTTTGGAGTGGTAGAAA  ACGACCTCAACGCACAGTA  CAAACTGGTGCTCAAGGC  AGTCTGATCACCAGTTTGCTG  CCAGCCGCCCAGTCTAATCA  GGACTGGTCTTTCTATCTCTTGT |
