# Supplementary material for: Induction of p53 Phosphorylation at Serine 20 by Resveratrol Is Required to Activate p53 Target Genes, Restoring Apoptosis in MCF-7 Cells Resistant to Cisplatin
Source: Nutrients. 2018 Aug 23;10(9):1148. doi: 10.3390/nu10091148 (PMC6163170; doi:10.3390/nu10091148)
Supplement: Supplementary file 1 [file nutrients-10-01148-s001.zip › Figure Supplementary Caption.docx]

**Figure S1.** Down-regulation of p53 in MCF-7 and MCF-7_R_ cells by shRNA. (A) Cells were stably transfected with shRNA sequences against p53 (p53-shRNA) or non-effective scrambled sequence as control (Ctrl-shRNA); Cells were treated with CDDP (6 μM) for 48 h to stimulate p53 expression and p53 contents were assessed by western blot using antibody against total p53 (DO-1). Non-transfected C33-A and MCF-7_R_ cells without CDDP treated only with DMSO vehicle were employed as control of normal unstimulated p53 expression. (B) Densitometric analysis of p53 after β-actin normalization. Results were presented as mean of three independent experiments ± SD. ****P*<0.001 by one-way ANOVA.

**Figure S2.** Transfected cells with Ctrl-shRNA are sensitive to Resv. Ctrl-shRNA transfected cells were treated for 48 h with indicated CDDP concentrations with or without Resv (100 μM). Cell viability was tested by MTT assay. Each data point is the mean of three independent experiments ± SD. The IC50 values for CDDP were calculated and shown in the box.
