# Supplementary figures and images for: Induction of p53 Phosphorylation at Serine 20 by Resveratrol Is Required to Activate p53 Target Genes, Restoring Apoptosis in MCF-7 Cells Resistant to Cisplatin
Source: Nutrients. 2018 Aug 23;10(9):1148. doi: 10.3390/nu10091148 (PMC6163170; doi:10.3390/nu10091148)

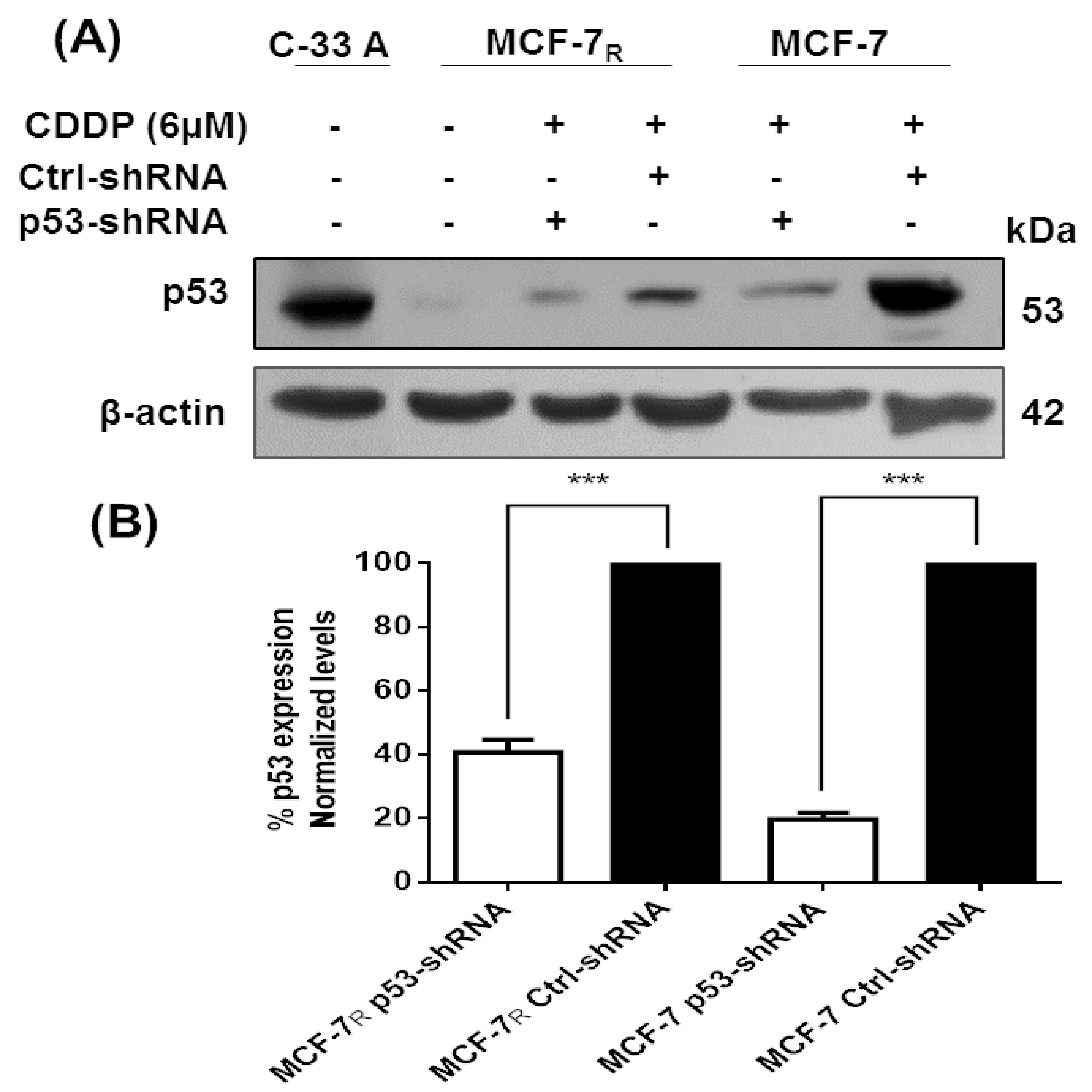

Supplement: Supplementary file 1 [file nutrients-10-01148-s001.zip › Figure S1.tiff]

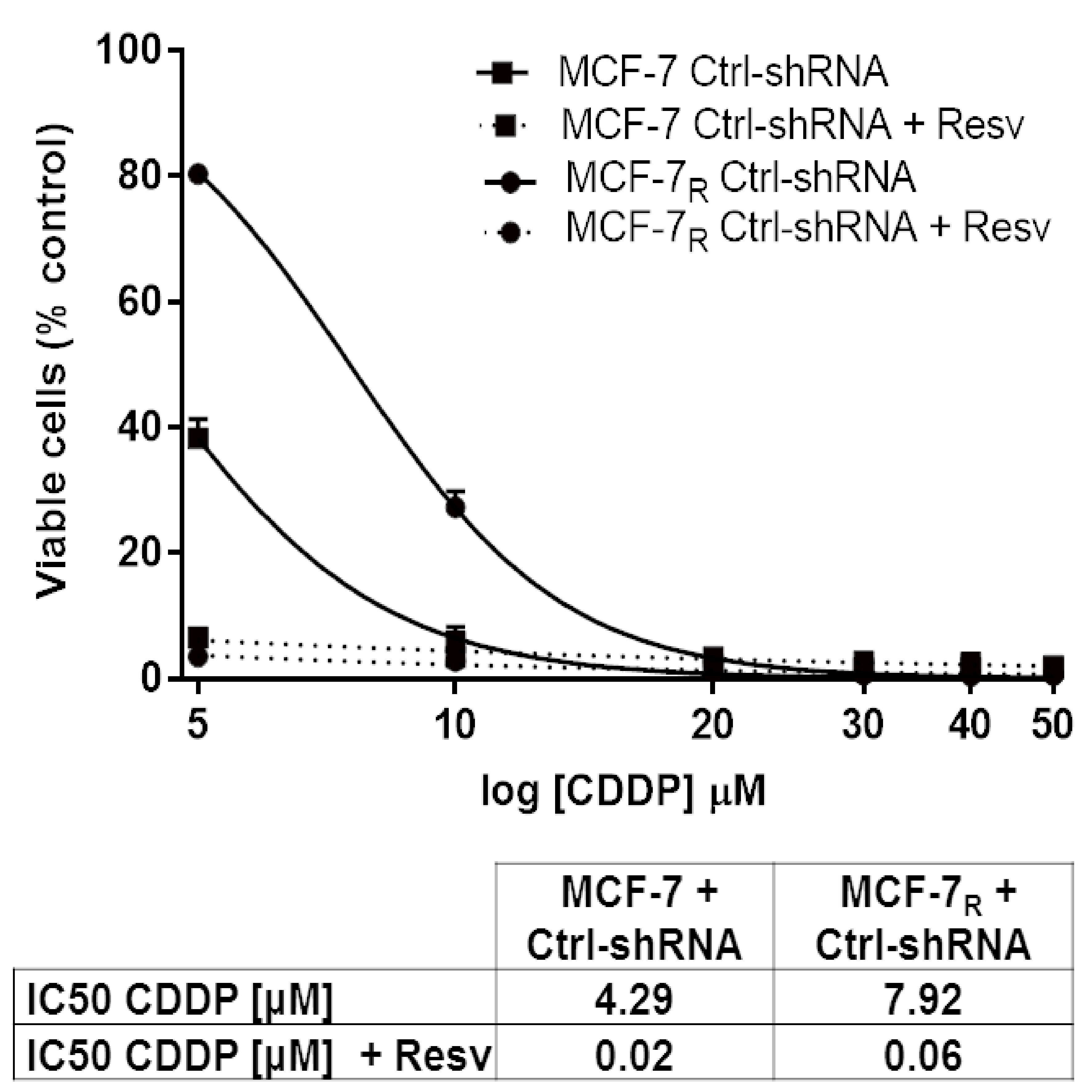

Supplement: Supplementary file 1 [file nutrients-10-01148-s001.zip › Figure S2.tiff]
